# refseqR: an R package for common computational operations with records on RefSeq collection

**DOI:** 10.1093/bioadv/vbae122

**Published:** 2024-08-21

**Authors:** Jose V Die

**Affiliations:** Department of Genetics—ETSIAM, University of Cordoba, Córdoba, 14071, Spain

## Abstract

**Summary:**

We introduce refseqR, an R package that offers a user-friendly solution, enabling common computational operations on RefSeq entries (GenBank, NCBI). The package is specifically designed to interact with records curated from the RefSeq database. Most importantly, the interoperability and integration with several Bioconductor objects allow connections to be applied to other projects.

**Availability and implementation:**

The package refseqR is implemented in R and published under the MIT open-source license. The source code, documentation, and usage instructions are available on CRAN (https://CRAN.R-project.org/package=refseqR).

## 1 Introduction

The Reference Sequence (RefSeq) collection at the National Center for Biotechnology Information (NCBI) provides a comprehensive, non-redundant, well-annotated set of sequences, including genomes, transcripts, and proteins. At the time of writing, the RefSeq project contains over 60 million transcripts and 320 million protein sequences. Among the distinguishing features, RefSeq exhibits format consistency and undergoes ongoing active curation by NCBI staff and collaborating groups (Sayers *et al.* 2022). RefSeq can be accessed through the NCBI FTP site (ftp.ncbi.nlm.nih.gov), as well as via two search and retrieval systems: BLAST, which conducts sequence-based searches, and Entrez, which performs natural language-based searches. The Entrez system comprises 39 molecular and literature databases, offering access through a search interface with powerful options for constructing precise searches and efficiently managing results. In addition, programmers can access RefSeq (and the whole GenBank database), using the Entrez Programming Utilities (E-Utilities), the public API to the Entrez system (https://eutils.ncbi.nlm.nih.gov/).

As biology evolves into a more data-centric field, computational thinking and computational methods are emerging as key aspects for achieving an in-depth understanding of modern biology. Among the programming languages favored by biologists, Entrezpy (Python library; Buchmann and Holmes 2019) and rentrez (R package; Winter 2017) are specifically designed to interact with E-utilities, offering comprehensive functions that cover the entire API. Complementary to these resources, and specifically in R, some packages from the Bioconductor project (e.g. MeSHSim; Zhou *et al.* 2015) or available from CRAN (e.g. RISmed; Kovalchik 2021) take advantage of the Eutils API to perform specific tasks.

Here, we described refseqR, made available in the R programming language, which provides a convenient framework to handle biological sequences hosted by the RefSeq collection. refseqR deals with the flow of genetic information within a biological system, allowing directional flows from gene locus collected as gene records, to transcripts and protein sequences curated from the RefSeq database, as well as other combinations among sequences of these molecules (Table 1). refseqR requires the end user to be familiar with the R programming language, but only at an elementary user level, as it eliminates the need for the user to be proficient and verbose with the functions that communicate with the server-side programs of E-Utilities for querying and downloading datasets from the nucleotide or protein databases. Although not yet published, some versions of the functions implemented here have been previously applied in our downstream applications, including sequence annotation, gene family characterization, and marking specific genes associated with agronomic traits (Die *et al.* 2018; Aguilar-Benitez *et al.* 2020; Carmona-Molero *et al.* 2021).

**Table 1.** Summary of functions available in refseqR.^a^

Function	Gene	Transcript	Protein
refseq_description	×	×	×
refseq_fromGene	×		
refseq_GeneID		×	×
refseq_CDScoords		×	
refseq_CDSseq		×	
refseq_RNA2protein		×	
refseq_RNAfeat		×	
refseq_protein2RNA			×
refseq_AAlength			×
refseq_AAmolwt			×
refseq_AAseq			×

a The availability of gene transcript or protein identifiers for each function is represented by a mark (×).

## 2 Implementation and functionalities

refseqR is a framework of common computational operations working with RefSeq entries. The functions have a consistent naming scheme. All functions in refseqR start with refseq_ and take a character vector as the first argument that represents a record identifier.

Following the Central Dogma of molecular biology from any gene record, the function refseq_description provides the sequence description from a gene accession. However, the function is also implemented for identifiers of transcripts or protein sequences. The function refseq_fromGene takes the GeneID identifier as its first argument and returns the corresponding transcript or protein id., as specified by the second argument. Although refseq_fromGene, like some other functions in the package, is implemented for a single identifier, its functionality over multiple identifiers, including large-scale operations, is guaranteed through the application of a split-apply-combine strategy. The available package documentation provides examples to illustrate this point. Depending on the function, available accessions in refseqR include RefSeq models with the prefixes XM_ (mRNA), XR_ (non-coding RNA), and XP_ (protein), which are produced either by NCBI’s genome annotation pipeline or from computationally annotated submissions to the INSDC. Available accessions may also include their subsequently curated RefSeq records with NM_, NR_, or NP_ accession prefixes.

Next, a number of operations are implemented for the mRNA molecule. For instance, refseq_GeneID returns the gene symbol identifier from a single mRNA accession and is also implemented for a protein identifier. Another function, refseq_CDScoords, parses an mRNA accession and extracts the coding sequence coordinates, identifying the genomic region for the 5′UTR and/or 3′UTR if present. On top of that function, refseq_CDSseq operates by parsing single or multiple mRNA IDs and extracting the nucleotide coding sequences into a DNAStringSet object (Pagès *et al.* 2024). The ability to parse accessions in GenBank format and extract the sequences into existing Bioconductor objects facilitates the interoperability and integrative analysis of data from different experiments (Huber *et al.* 2015). Finally, refseq_RNA2protein returns the corresponding protein ID from the specified RNA accession.

Concluding with the suite of functions designed for managing protein accessions, refseq_AAlength returns the amino acid length of the sequence, while refseq_AAmolwt provides the molecular weight in Daltons. refseq_AAseq functions analogously to refseq_CDSseq, parsing single or multiple protein identifiers to extract the amino acid sequences into a BString object. In line with the flow from RNA to protein databases and vice versa, refseq_protein2RNA returns the corresponding mRNA identifier from the specified protein accession.

## 3 Concluding remarks

Data literacy skills have become central to the biology curriculum. Molecular biologists lacking a foundation in programming, who must navigate vast datasets, encounter a formidable learning curve. However, by leveraging a suite of wrappers built upon top-tier packages and libraries, the complexity of these tasks can be greatly reduced. refseqR is an R package that offers a user-friendly solution, enabling common computational operations on GenBank databases with minimal coding expertise. Tailored for seamless interaction with records sourced from the RefSeq database, its integration with various Bioconductor objects ensures interoperability and facilitates connections between datasets, with versatile applicability across diverse projects.

## Data Availability

The data underlying this article are available in CRAN, at https://CRAN.R-project.org/package=refseqR [https://doi.org/10.32614/CRAN.package.refseqR].
